# Provenance differences and factors influencing transpiration of *Cunninghamia lanceolata* in a common garden experiment

**DOI:** 10.3389/fpls.2025.1515534

**Published:** 2025-02-24

**Authors:** Tingyu Xu, Xiang Niu, Bing Wang

**Affiliations:** ^1^ Ecology and Nature Conservation Institute, Chinese Academy of Forestry, Beijing, China; ^2^ Key Laboratory of Forest Ecology and Environment of National Forestry and Grassland Administration, Beijing, China; ^3^ Dagangshan National Key Field Observation and Research Station for Forest Ecosystem, Xinyu, China

**Keywords:** forest water consumption, sap flow, common garden, provenances, meteorological factors

## Abstract

Tree transpiration is a key component of forest evapotranspiration, and sap flow monitoring is the primary way to study tree canopy transpiration and water consumption. However, provenance differences in transpiration and the unique responses to environmental factors are not well understood. We measured the sap flow and calculated the canopy evapotranspiration (Ec) of 15 Chinese fir provenances from five provinces in a common garden and monitored soil moisture and meteorological variables between September 2020 and September 2022. Mean daily Ec of the provenances from Guangxi (GX), Sichuan (SC), Anhui (AH), Yunan (YN), and Zhejiang (ZJ) were 1.31 ± 0.99 g·d^−1^, 1.59 ± 1.18 g·d^−1^, 1.62 ± 1.43 g·d^−1^, 1.41 ± 1.01 g·d^−1^, and 1.48 ± 1.13 g·d^−1^ during the study period, respectively. The mean daily Ec of Guangxi, Sichuan, Anhui, Yunan, and Zhejiang provenances exhibited significant differences (*p* <0.01). Overall, the Ec of these provenances was high from June to August. Soil moisture had different effects on the Ec of the provenances. The provenances from Zhejiang, Sichuan, and Anhui showed higher Ec values when REW <0.4 than REW≥0.4 conditions, but the Ec of Guangxi and Yunnan provenances showed no significant differences under the two conditions. When the soil was relatively moist, Ec of the provenances was mainly influenced by Rs and VPD. When the soil was relatively dry, the main influencing factors were the Ta and VPD. Overall, our findings revealed different provenance-specific responses of Ec to biophysical factors, providing valuable insights for the selection of superior provenances of Chinese fir from the perspective of water use in the context of a changing climate.

## 1 Introduction

Global climate change has significantly affected the composition, structure, and functions of terrestrial ecosystems (Weiskopf et al., 2020). China has the world’s largest planted forest area (Cheng et al., 2024). Planted forests in many regions are expected to experience increased frequency and intensity of seasonal drought and extreme precipitation episodes (Sinacore et al., 2019; Taylor et al., 2017), which may considerably affect the structural function and sustainability of forest ecosystems (Hammond et al., 2022). The risk of widespread decline and death in forests remains high (Choat et al., 2018), and scientific management measures must be implemented to prevent this as soon as possible (Manrique-Alba et al., 2022). Plant water use characteristics are critical for sustaining vegetative growth and productivity, particularly in the context of global climate change (Hiroki et al., 2021; Zhao et al., 2022).

Transpiration is the primary plant water consumption pathway and an important component of the water balance in forest ecosystems (Liu et al., 2020, 2021). It reflects the balance of rainfall–transpiration–infiltration, and is crucial for the establishment of water balance mechanisms and regional sustainable water resource management plans (Meusburger et al., 2022). Through photosynthesis, plant roots absorb water from the soil and transport it to the canopy through the stem (Cai et al., 2022). Water finally dissipates through the leaf stomata, which is key in connecting the soil–plant–atmosphere continuum (SPAC) (Deng et al., 2017; Kannenberg et al., 2023). Stem sap flow characterizes water consumption and reveals the characteristics of the specific transpiration response to meteorological conditions and the soil moisture environment (Wan et al., 2023; Li et al., 2023). Thermal dissipation probe technology (TDP) has been used to monitor the sap flow of tree sapwood, which is an important means of exploring changes in water consumption (Pasqualotto et al., 2019). Many studies have suggested that meteorological factors play a decisive role in tree transpiration (Li et al., 2024; Deng et al., 2021). Factors such as air vapor pressure deficit (VPD) and solar radiation (Rs) reflect the atmospheric evaporation demand and are the main driving forces of canopy transpiration (Sun et al., 2021; Chen et al., 2022a). Rs directly affects the opening and closing of leaf stomata, whereas VPD is the driving force of tree transpiration (Pieruschka et al., 2010; Fernandez et al., 2009). Integrating variables that characterize the changes in Rs and VPD comprehensively reflects the response characteristics of stem sap flow to these factors (Lyu et al., 2022). Whitley et al. (2008) found that, in addition to Rs and VPD, incorporating soil moisture into forest water consumption models can accurately estimate water consumption at the forest stand scale. Navarro et al. (2020) demonstrated that the photosynthetic photonflux density, VPD, soil temperature, air temperature, and soil water content all influence Ec. Wang et al. (2020) proposed a new temperature stress function to fit the entire range of sap flow. The response of plant water use to climate change is partly related to the tree species composition of the forest ecosystem (Christoph et al., 2023). In addition, plants respond to environmental conditions by regulating their specific hydraulic systems and leaf stomata (Chen et al., 2022b). Different species have different water-use strategies and transpiration rates. For example, isohydric plants reduce water transpiration losses by closing stomata under water stress, whereas anisohydric plants maintain partial opening of stomata and higher transpiration rates even under water stress (Hochberg et al., 2018). Recent extreme heat events directly and severely affect tree health (Li et al., 2022). In the context of climate change, the effectiveness of water use strategies is important for tree survival. It is necessary to understand how transpiration in different tree species responds to environmental changes.

*Cunninghamia lanceolata* is the main timber forest species mentioned in the “National Reserve Forest Construction Plan (2018–2035)” of the “Hunan Hubei Jiangxi Luoxiao Mountain National Reserve Forest Construction Project,” which has a cultivation history of over 1,000 years in China. The ninth forest resource inventory data show that the total area of *C. lanceolata* plantations has reached 990.20 × 10^4^ hm^2^, accounting for 29% of the total planted forest area, with a storage volume of 7.55 × 108 m^3^ (SFA, 2019). Maintaining the long-term productivity of *C. lanceolata* plantations is of great significance for improving the stability of regional ecosystems, quality of regional forests, and regional ecological environment (Tanay et al., 2023). However, changes in precipitation patterns caused by climate change and extreme weather pose a serious threat to improving *C. lanceolata* productivity in subtropical China (Zhang Y. et al., 2023). Hence, to better formulate scientific water management policies in the context of climate change, possible species-specific water-use regulations for *C. lanceolata* must be clarified.

The *C. lanceolata* provenance experiment conducted in the 1970s as a large-scale field control test site for studying the long-term response of plants to climate change was useful for analyzing the geographical provenance effects and providing a basis for selecting provenances (Hong, 1994). Studies have reported that genetic effects explain 50%–70% of the variations in *C. lanceolata* productivity (Wu et al., 2021), and it is necessary to reveal the mechanism of productivity from the perspective of adaptation of the provenance to a changing environment. Significant differences in genetic composition, morphology, adaptability, growth, resistance to temperature, humidity, and other factors in *C. lanceolata* plantations have been observed during evolution (Hong, 1994). *C. lanceolata* distributed between 25°N and 28°N exhibited better growth rates than those at other provenances (Wu et al., 2021). Experiments conducted in common gardens have been limited to research on growth characteristics and functional traits (Klisz et al., 2019), but the regulation of plant growth is crucial under changing climate conditions (Jiao et al., 2021; Zhang et al., 2023), while there are few reports on water consumption in common garden experiments. At present, research on sap flow and its response to environmental conditions is being conducted both domestically and internationally for different vegetation types (Steppe et al., 2015). However, further research is required to explain the adaptability of provenances to the environment from the perspective of water use in the context of climate change. Therefore, we compared and determined the water use relationships of the provenances in a common garden of *C. lanceolata*, to reveal the regulatory mechanism of plant water consumption and provide scientific water management for plantations in subtropical areas. Specifically, we hypothesized that (1) the canopy transpiration of *C. lanceolata* provenances would be significantly different, (2) the response of canopy transpiration to soil water conditions varies for different provenances, and (3) the response of canopy transpiration to environmental factors varies for different provenances because provenances will exhibit different water use strategies during the dry and wet seasons.

## 2 Materials and methods

### 2.1 Study site

**Figure 1:**
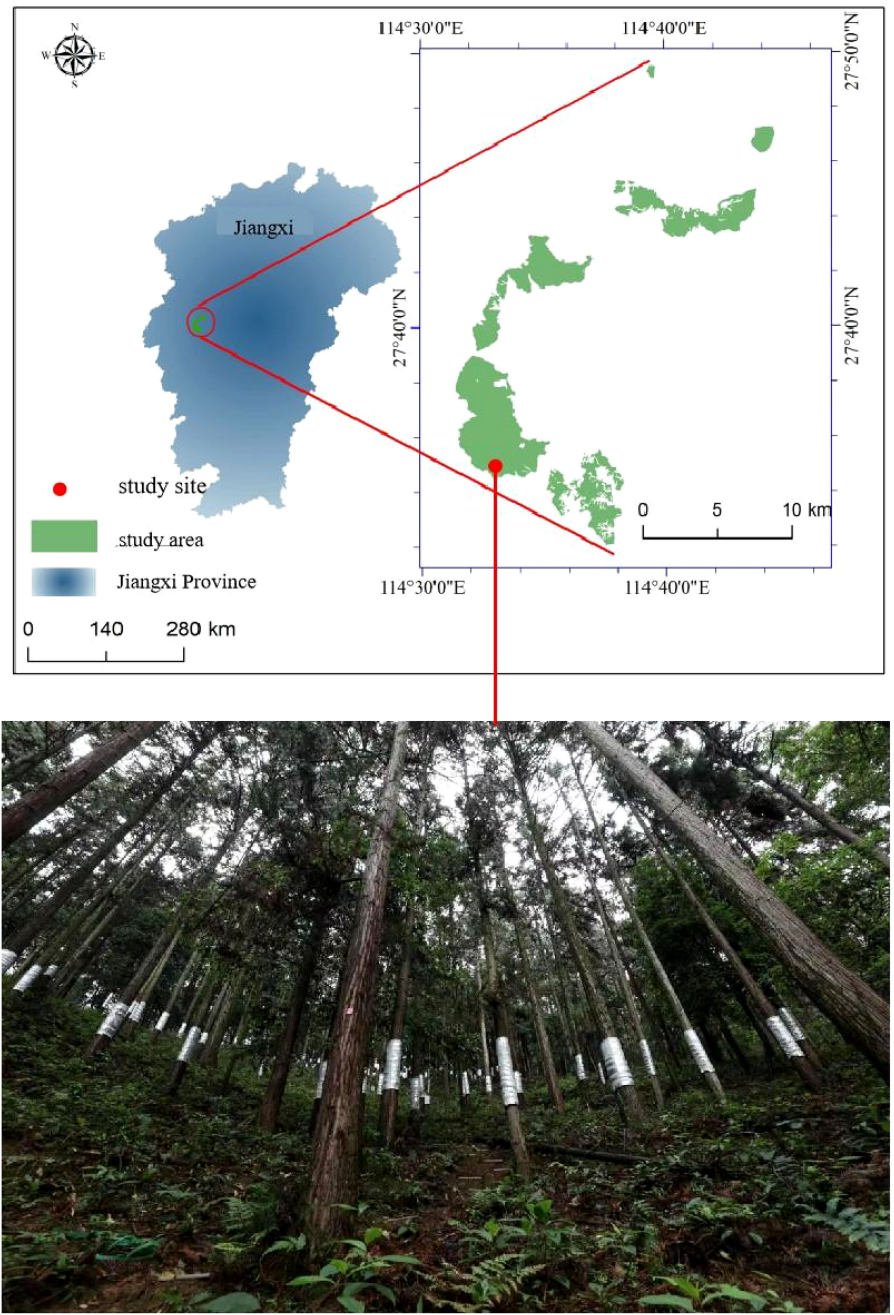
The location of study area.

This study was conducted in Dagangshan, JiangXi Province (**Figure 1**), China (114°31′23″E–114°44′31″E, 27°32′2″N–27°49′31″N). The mean annual air temperature and precipitation are 15.8°C and 1,600 mm, respectively. Most of the precipitation occurs from April to June, accounting for 45% of the annual precipitation.

In 1981, the Chinese Academy of Forestry conducted a geographical provenance experiment on Chinese fir in Dagangshan, which established a common garden containing 183 Chinese fir provenances. In this study, we selected 15 provenances (**Table 1**) from five provinces growing in the common garden in Dagangshan for 41 years under the same climatic conditions with consistent slope orientation, soil, and altitude conditions, located in adjacent groups, during the provenance experiment design to investigate the transpiration characteristics of Chinese fir from different regions.

**Table 1:** Basic characteristics of the provenances.

Province	Provenance	DBH (cm)	Sapwood area (cm^2^)
Guangxi(GX)	Guigang, Guangxi	21.0	47.9
	Gongcheng, Guangxi	24.4	59.8
	Lingchuan, Guangxi	32.3	90.5
Sichuan(SC)	Hongya, Sichuan	22.3	52.3
	Pengxian, Sichuan	18.8	40.6
	Nanhong, Sichuan	23.2	55.5
Anhui(AH)	Taihu, Anhui	23.4	56.2
	Jinzhai, Anhui	13.7	25.4
	Xiuning, Anhui	23.6	56.9
Yunnan(YN)	Zhenxiong, Yunnan	18.2	38.7
	Xichou, Yunnan	18.8	40.6
	Maguan, Yunan	21.3	48.9
Zhejiang(ZJ)	Lishui, Zhejiang	20.4	45.8
	Longquan, Zhejiang	32.0	89.4
	Yunhe, Zhejiang	23.8	57.6

**Figure 2:**
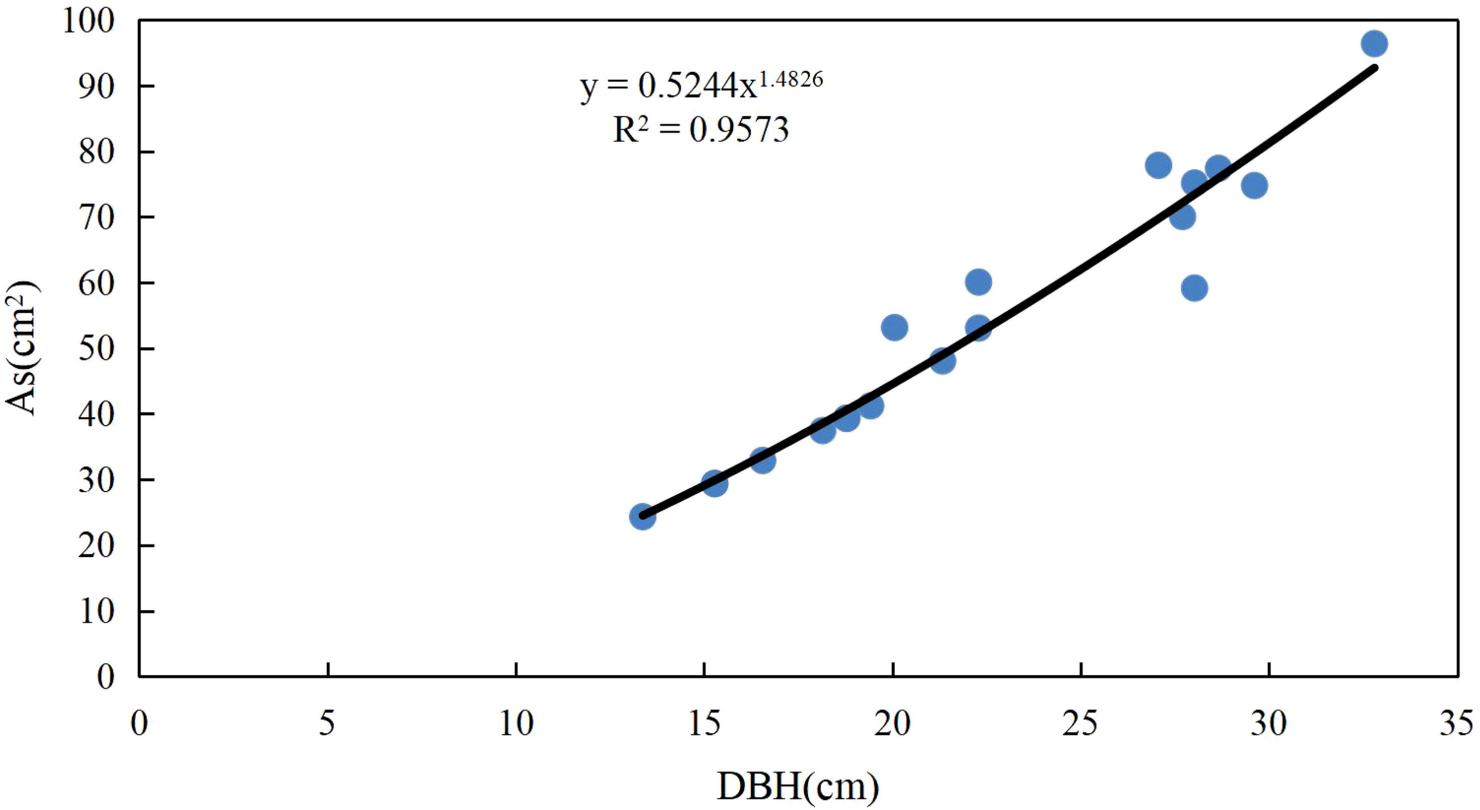
The relationship between sapwood area and diameter at breast height.

The area of Chinese fir sapwood (As) was indirectly measured using the growth cone method, and 20 Chinese fir trees with good growth status and different diameters at breast height were selected from the plot. During the growing season, sapwood samples were collected from the diameter at breast height using a growth cone drill, and the length of the sapwood was measured using a Vernier caliper to calculate As (**Figure 2**).

### 2.2 Monitoring environmental factors

Meteorological factors, including precipitation (P, mm), air temperature (Ta, °C), relative air humidity (RH, %), solar radiation (Rs, w·m^−2^), and wind speed (Ws, m·s^−1^), were measured by an automatic weather station (Campbell Scientific, Inc., Logan, UT, USA) every 10 min in an open field approximately 100 m away from the study plot. The vapor pressure deficit (VPD, kPa) was calculated using Ta and RH using Equation 1 (Campbell and Norman, 1998).


(1) $$VPD=0.611\times (1-RH)\times \exp\left(\frac{17.502\times T_{a}}{T_{a}+240.97}\right)$$

The soil volumetric water content(VWC) in the different layers of (0 cm–20 cm, 20.1 cm–40 cm, 40.1 cm–60 cm, 60.1 cm–80 cm, and 80.1 cm–100 cm) was monitored at a single location close to the sap flow-measured in the Chinese fir plot using TDR 100 soil moisture sensors(Campbell Scientific, Inc., Logan, UT, USA). Data were recorded using a CR1000 logger (Campbell Scientific, Inc., Logan, UT, USA) every 10 min. The relative extractable water (REW) in the soil layer was calculated using Equation 2:


(2) $$REW=\frac{VWC_{a}-VWC_{w}}{VWC_{c}-VWC_{w}}$$

where VWCa (%), VWCw (%), and VWCc (%) are the averages of the actual VWC, soil wilting moisture, and soil field capacity in the 0 cm–100 cm soil layer, respectively. In a previous study, stand transpiration was found to be more affected by soil moisture when REW <0.4 (Chen et al., 2022a). Thus, soil water availability can be classified into two categories: no soil water stress when REW ≧0.4, and water stress when REW <0.4.

### 2.3 Sap flow measurement and transpiration calculation

This study focused on 15 different Chinese fir provenances (with good growth conditions, consistent environment, minimal difference in diameter at breast height, and no pests or diseases). Continuous measurements were performed using a thermal dissipation liquid flow measurement system (TDP-100, China) between 1 September 2020 and 30 September 2022. To prevent the effects of direct sunlight heating on the sensor, a location on the shaded surface of a tree where the sensor was installed was chosen. By shaving off the dried bark, a rectangular space of approximately 3 cm × 6 cm was cleared to avoid damaging the living tissue of the tree. The sensors were installed 1.3 m from the ground to avoid heat gradients caused by cold stem flow coming out of the soil, and one-quarter spherical foam was installed on each side of the TDP probe to protect the sensor wire from twisting pressure and to increase thermal insulation around the probe. Reflective foam, foam balls, and TDP installation parts were used to wrap the outermost layers of the trees. Data from the TDP sensors were collected every 10 min using CR1000 (Campbell Scientific Inc., Logan, UT, USA). Sap flow was calculated using Equation 3 proposed by Granier (1987):


(3) $$V=0.0119\times {\left[\left(\text{dT}M-\text{d}T\right)/\text{dT}\right]}^{\;1.231}$$

where dT is the temperature difference between the two probes (°C), dTM is the maximum dT value within a day, and V is the sap flow rate (g·m^-2^·s^−1^).

The transpiration water consumption of the trees was determined based on the sap flow rate and the sapwood area of the trunk, which can be calculated using Equation 4:


(4) $$E_{\text{c}}=A\text{s}\times V\times 3600\times 24$$

where Ec is the water consumption within a day (, g·d^−1^) and As is the sapwood area of the trunk (cm^2^).

### 2.4 Statistical analysis

The Ec of the provenances was studied by province. The provenances from each province were collected, and the mean value was calculated at different timescales. To avoid the possible uncertainty generated by wet canopies and the atmosphere (Chen et al., 2020), data on rainy days were removed when analyzing the responses of stand transpiration to environmental factors (a total of 404 days with 0 rainfall were used). Before the analysis, homogeneity of variance and normality tests were performed on the data. If the prerequisites for analysis of variance were not met, Kruskal–Wallis tests were performed to examine the differences in the Ec among provenances, and the Mann–Whitney U test was performed to examine the differences in transpiration under different soil moisture conditions of the provenances.

We used path analysis to quantify the direct and indirect effects of environmental factors on the Ec of the provenances. All explanatory variables (VPD, Rs, and Ta) were assumed to impact the predicted variables (Ec) before the path analysis. The direct and indirect effects of each factor were quantified by standardized path coefficients, which were calculated using the maximum likelihood method, and the total effects were the sum of the direct and indirect effects. The final model structure was determined when the root mean square error of approximation (RMSEA) was <0.05, and the goodness of fit index (GFI) was >0.95. Path analysis was performed using AMOS (version 26.0; IBM SPSS, Chicago, IL, USA).

## 3 Results

### 3.1 Climatic and soil moisture characteristics

**Figure 3:**
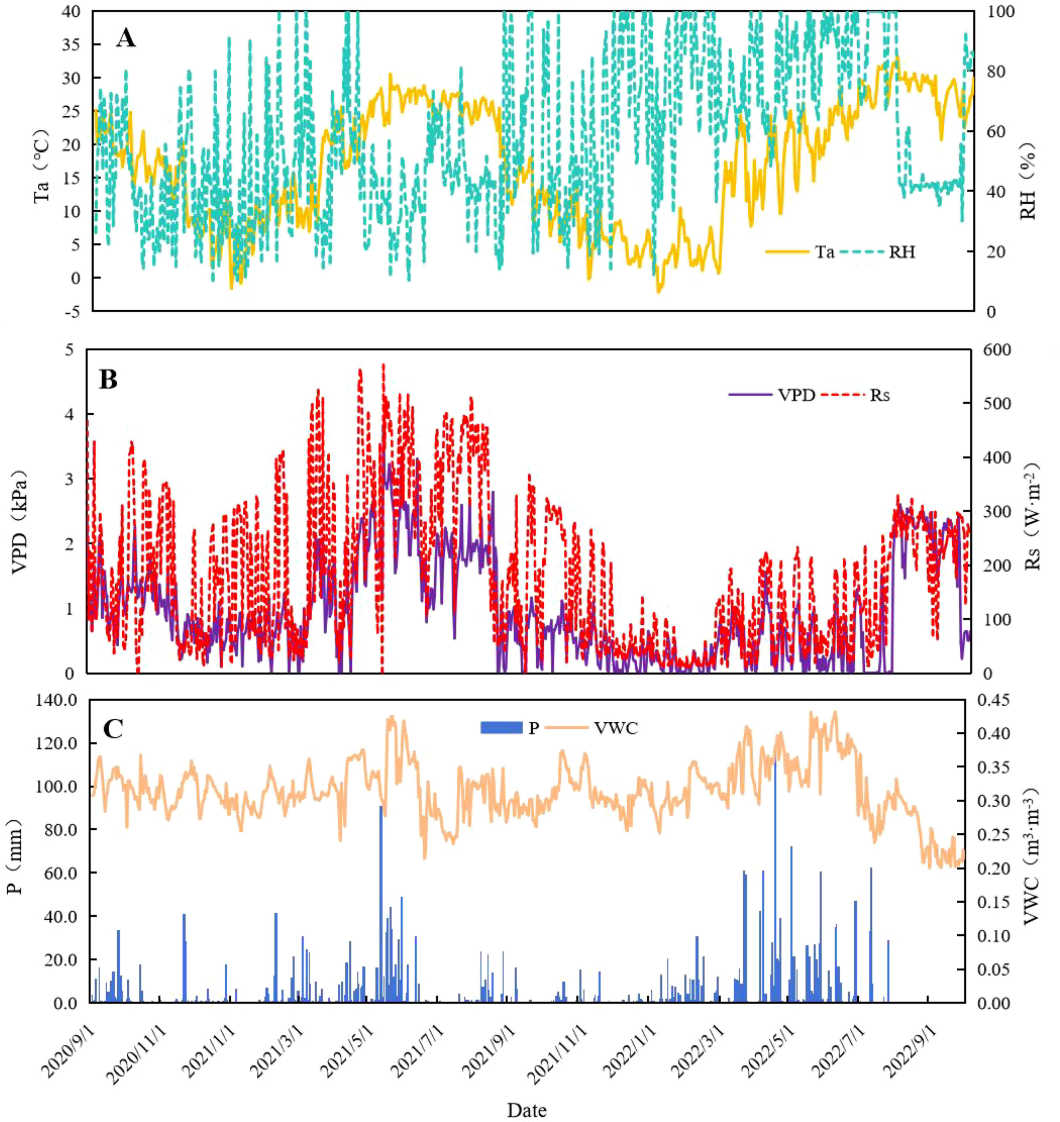
Variations in daily air temperature (Ta), relative air humidity (RH) **(A)**, solar radiation (Rs, w·m^−2^), vapor pressure deficit (VPD) **(B)**, precipitation (P), and soil volumetric water content (VWC) **(C)** during the study period.

The daily variations in meteorological factors are summarized in **Figure 3**. The daily mean VPD, Rs, Ta, RH, and Ws values were 0.95 kPa ± 0.79 kPa, 181.07 W·m^−2^ ± 136.97 W·m^−2^, 16.77 °C ± 9.02 °C, 56.18% ± 38.66%, and 0.49 m·s^−1^ ± 0.18 m·s^−1^, respectively. Overall, VPD and Rs reached their maxima in May 2021. Ta was the highest in July and lowest in January. There were 145 days with RH ≥90%. The P was mainly distributed in April and May. P was ≤10 mm in August and September 2022. The daily mean VWC was 0.313 m^3^·m^−3^ ± 0.043 m^3^·m^−3^. Variations in VWC were generally similar to those in P, which reached the highest values in May and June.

### 3.2 Differences in water consumption of the provenances

**Figure 4:**
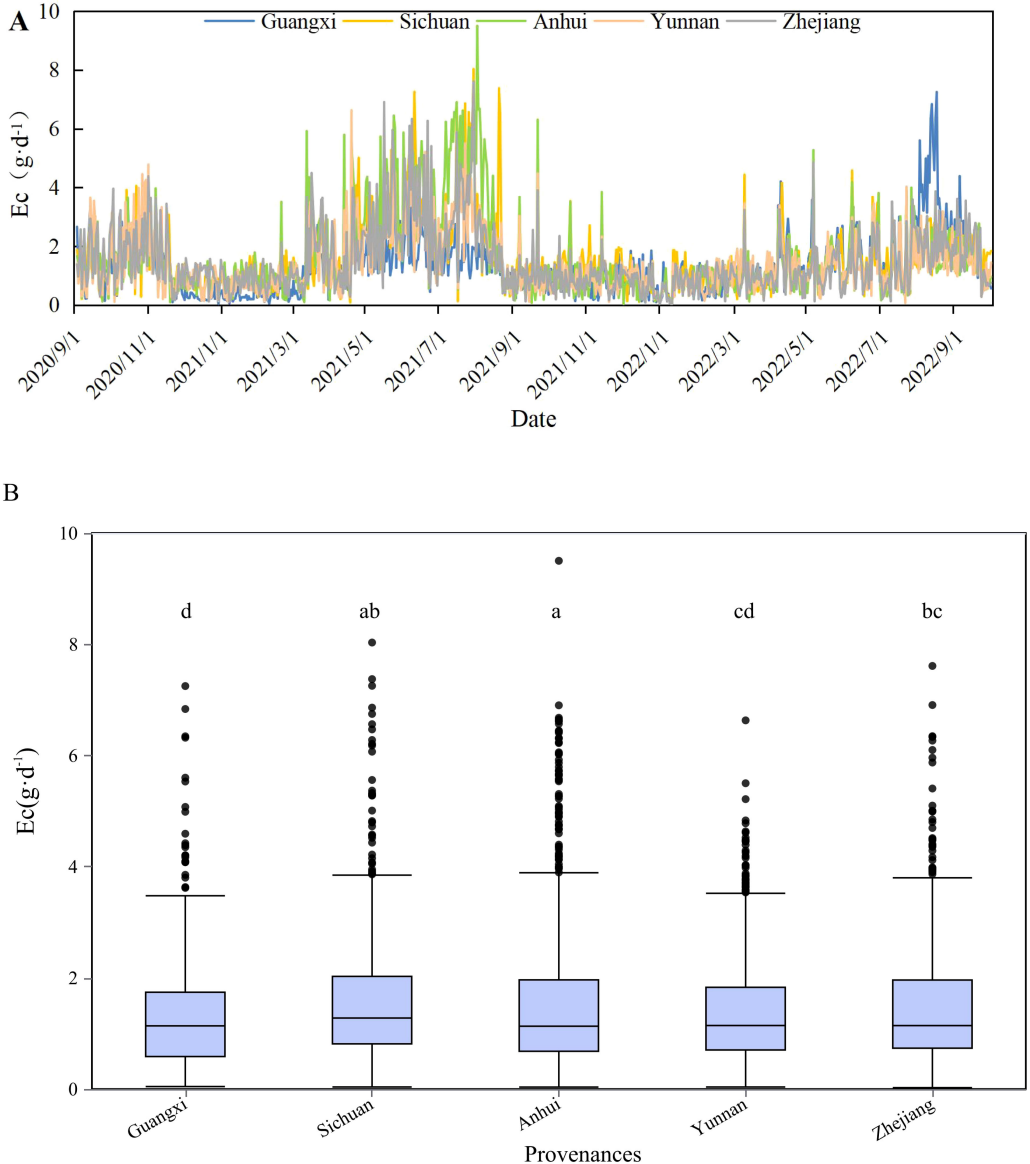
Daily variations **(A)** in the Ec of different provenances and distribution of Ec of different provenances **(B)**. Different letters indicate significant differences among the provenances (*P <*0.05).

The mean daily Ec values of the provenances from Guangxi, Sichuan, Anhui, Yunan, and Zhejiang were significantly different (*P <*0.01). The Ec were 1.31 g·d^−1^ ± 0.99 g·d^−1^, 1.59 g·d^−1^ ± 1.18 g·d^−1^, 1.62 g·d^−1^ ± 1.43 g·d^−1^, 1.41 g·d^−1^± 1.01 g·d^−1^, and 1.48 g·d^−1^ ± 1.13 g·d^−1^ during the study period, respectively. The Ec of Guangxi provenances was significantly lower than that of the other provenances (*P <*0.05). The Anhui and Zhejiang provenances did not show significantly different water consumption patterns (*P >*0.05). Overall, the Ec of the provenances was high from June to August. The Ec of the provenances from Guangxi reached its maximum value in August 2022, whereas the other four provenances reached a maximum in June 2021. All the provenances experienced low levels between December and February (**Figure 4**).

### 3.3 Differences in water consumption under different soil water conditions

**Figure 5:**
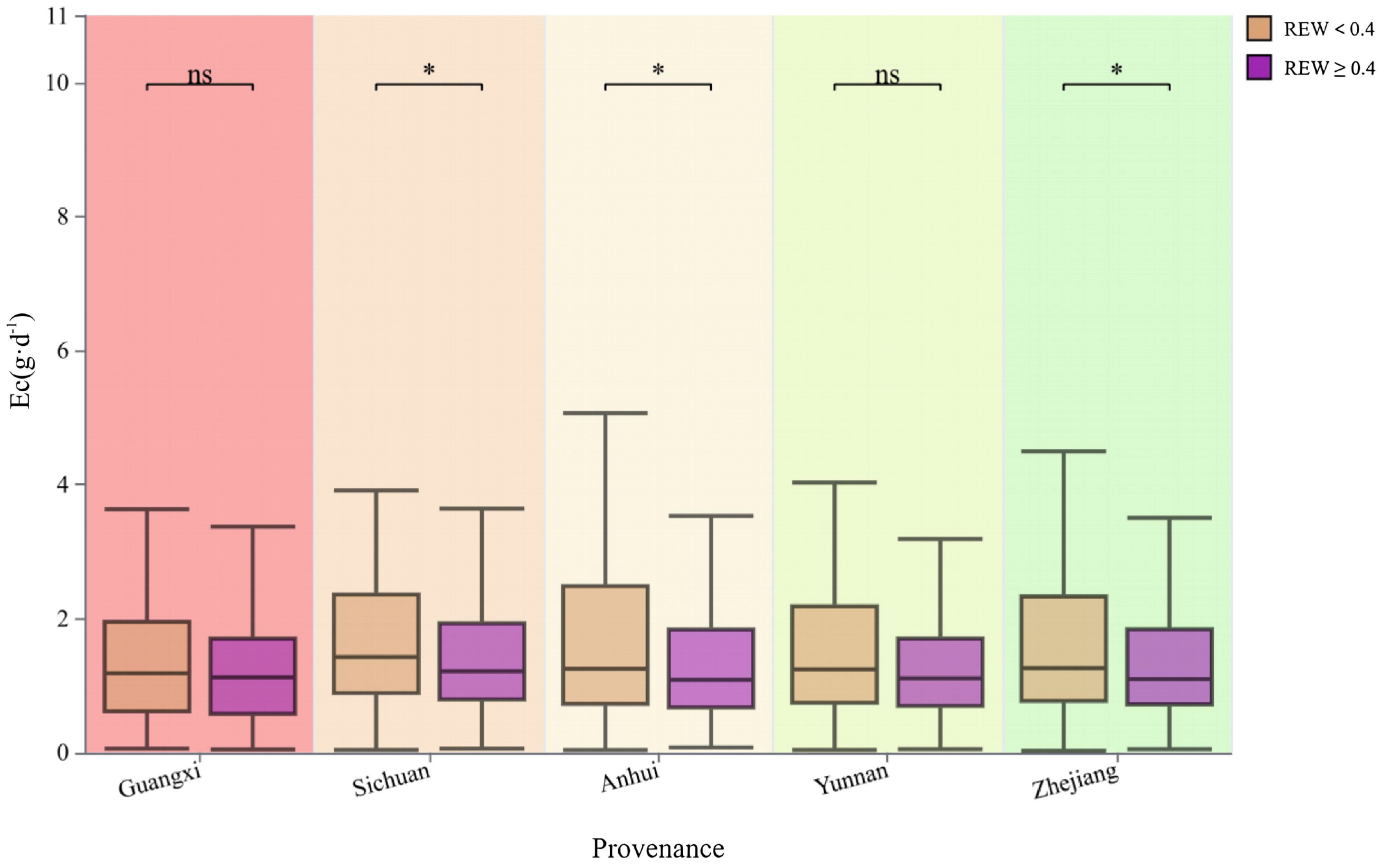
Provenance differences in Ec under different soil water availability conditions. NS, no significant difference (*P >*0.05); *, significant difference (*P <*0.05).

The soil moisture had different effects on the Ec of the provenances (**Figure 5**). Sichuan, Anhui, and Zhejiang provenances had significantly higher Ec values when REW <0.4 than REW >0.4. However, the difference in the Ec of the Guangxi and Yunnan provenances under the two conditions was not significant. Among them, the Anhui provenances showed the greatest difference between the two cases, with 18.19% higher Ec in REW <0.4 than REW≥0.4.

### 3.4 Provenance differences in the environmental responses of Ec

**Figure 6:**
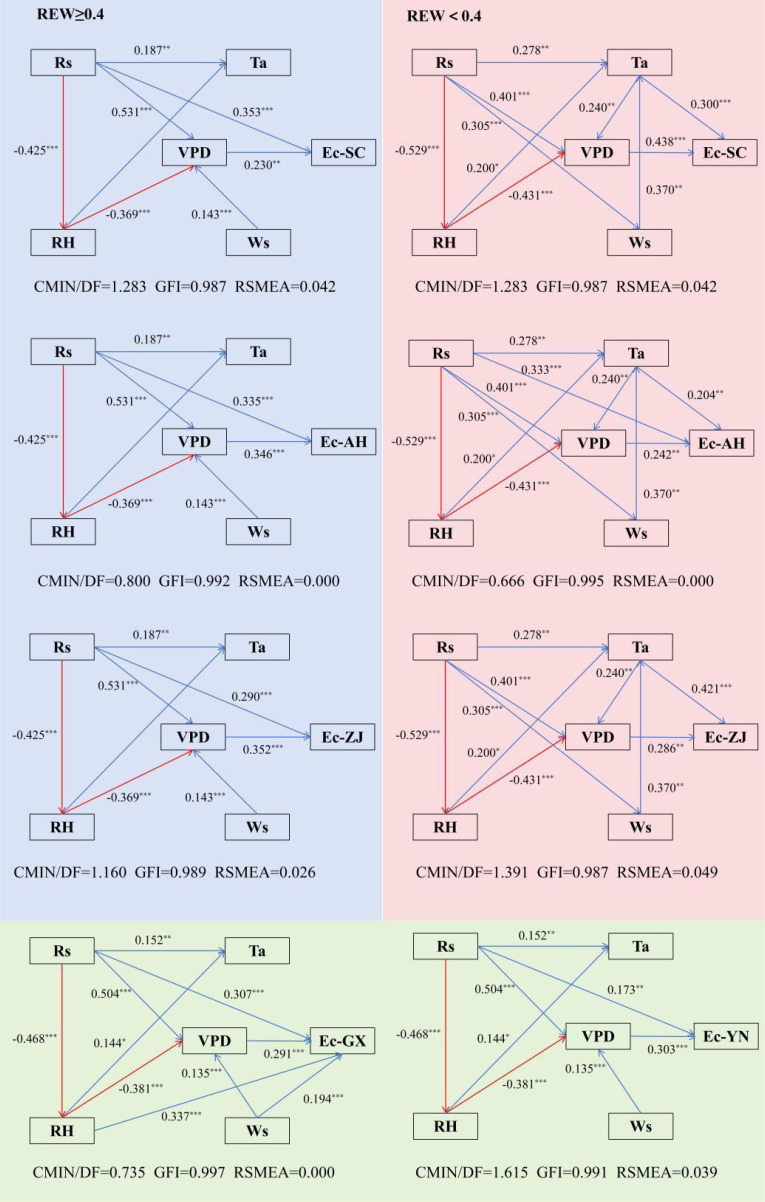
Direct and indirect effects of meteorological variables on Ec. The blue area is the analysis for REW ≥0.4 condition, the red area is the analysis for REW <0.4 condition, the green area is the analysis for the entire study period. Red and blue arrows represent significant positive and negative impacts, respectively. ^***^: p <0.001, ^**^: p <0.01.

Path analysis was conducted to assess the effects of meteorological factors on Ec under different REW conditions for provenances in Sichuan, Anhui, and Zhejiang. For provenances from Guangxi and Yunnan, we tested the meteorological influence on Ec throughout the study period (**Figure 6**). When REW was ≥0.4, the main factors affecting Ec were Rs and VPD (*p* <0.001). However, Ta and VPD become dominant influencing factors under REW <0.4 conditions (*p* <0.001), and only AH provenances also be affected by Rs under these conditions (*p* <0.001). The effectiveness of VPD on Ec diminished as REW decreased, except for SC provenance. Throughout the study period, the Ec of Guangxi provenances was influenced by Rs, VPD, Ws, and RH, while the impact factors for the Yunan provenances were limited to Rs and VPD. The effects of environmental factors on Ec varied among provenances.

## 4 Discussion

### 4.1 Ec of the provenances

Sap flux and sap flux density exhibited considerable variation across tree species. Many studies have shown that significant differences exist among species, attributed to physiological differences, such as DBH and lifestyle (Schoppach et al., 2021; Siddiq and Cao, 2018). In this study, the Ec of the provenances was significantly different (*P <*0.01) during the study period. The Ec of the Anhui provenances was relatively higher than that of the other provenances, whereas the Guangxi provenances exhibited a lower Ec. Tree species, size, and physiological characteristics influence tree transpiration (Jose et al., 2021). Wu et al. (2021) found that the growth rate of different provenances varies, leading to significant differences in tree height and DBH. Many studies have indicated that trees with a larger DBH typically have higher transpiration requirements, thus requiring higher sap flow rates to meet their water demands (Wang et al., 2023). However, in this study, the Guangxi provenance, which had the largest DBH, exhibited the lowest Ec. This phenomenon can primarily be attribute to the fact that DBH is not the sole factor affecting sap flow, such as crown size, as well as the number of branches and leaves, also play significant roles (Tfwala et al., 2019). The differences in transpiration among the different provenances may be related to their physiological mechanisms (Gao et al., 2021).The structure of the xylem directly affects the efficiency of water transmission. The size, quantity, and arrangement of xylem conduits influence both the speed and efficiency of the sap flow (Trueba et al., 2019). Species with larger diameters and a larger number of conduits usually exhibit higher tranpiration rates (Zhang et al., 2022). Furthermore, studies have demonstrated that stomatal density and the mechanisms governing stomatal opening and closing differ among provenances (Chen et al., 2023; Xu R. et al., 2023), which can affect transpiration rates and, consequently, overall water consumption. Differences in hydraulic conductivity among tree species are also critical factors affecting transpiration. Species with high hydraulic conductivity can maintain high sap flow rates under different conditions (Chen et al., 2022b). Provenances from low-rainfall areas tend to possess strong tolerance to diverse climatic conditions and employ more effective water conservation strategies, whereas provenances from high-rainfall areas may exhibit different adaptive strategies, resulting in differences in Ec among provenances. Wang et al. (2022) found that the response of provenances to temperature and precipitation can be divided into various geographic groups along longitudinal and latitudinal gradient, leading to distinct patterns in growth and transpiration. Additionally, studies have identified significant functional trait differences among provenances (Xu R. et al., 2023; Shou et al., 2024), which can influence resource acquisition and utilization, ultimately resulting in differences in growth or water consumption (Xu T. Y. et al., 2022). Moreover, water use efficiency varies among different provenances (Xu R. et al., 2023), contributing to significant differences in water consumption.

### 4.2 Differences in Ec under different soil water conditions

The results of this study indicated that the Ec of all provenances was higher when REW <0.4 than REW >0.4. This phenomenon can be attributed to the fact that high Rs and VPD facilitate transpiration pull and capillarity, enabling water uptake at the beginning of the drought (Justyna et al., 2022). Conditions with REW >0.4 often occurred the day after rainfall, during which RH was relatively high, VPD was relatively low, and the boundary layer was unsuitable for tree transpiration (Xu et al., 2020). Some studies conducted in arid regions have presented differing results compared with the present study. For instance, research in a semi-arid area has shown that a relatively low REW increases the degree of xylem embolism and reduces the gas exchange rate when plants experience drought, leading to a significant decrease in Ec (Lyu et al., 2022). Chen et al. (2022a) found that soil moisture significantly affects tree transpiration in semi-arid urban environments, where trees reduce transpiration during the dry season to minimize excessive water consumption. Conversely, other studies have indicated that these trees tend to have more stable water consumption in non-urban areas (Chen et al., 2023). Additionally, some tree species have been identified as insensitive to drought, suggesting that they possess a high adaptability to soil moisture deficiency (Du et al., 2011). Based on the present results, soil moisture may not be a vital factor affecting plantations in subtropical areas of China. Some studies have reached similar conclusions. The impact of soil moisture content on transpiration throughout the drought period was very low (Oogathoo et al., 2020; Song et al., 2020).Research in the subtropical regions of China has found that canopy transpiration of *Schima superba* is less limited by soil moisture during the rainy season, and the overall effect is mainly influenced by energy factors (Li et al., 2023). Even under dry conditions, these trees can maintain relatively stable transpiration rates, thereby enhancing water-use efficiency and ultimately promoting growth (Ouyang et al., 2022). Regarding Ec under different REW conditions, the range of Ec from the Anhui provenances was the highest, indicating that they adopted a progressive strategy with less sensitivity to stomatal regulation. Differences in soil moisture conditions did not significantly affect the transpiration of Guangxi and Yunnan provenances, likely because of their overall weaker transpiration.

### 4.3 Differences in environmental controls on the Ec among the provenances

The differences in transpiration among provenances are related to provenance-specific water use and sensitivity to environmental factors (Wang et al., 2019). Many studies have shown that physiological and environmental factors influence the plant Ec (Hayat et al., 2022; Ma et al., 2019). Meteorological conditions, particularly Rs and VPD, are the primary factors that control canopy transpiration (Oogathoo et al., 2020). Liu et al. (2023) found that there is a significant nonlinear relationship among Rs, VPD, REW, and LAI and Ec. Although VPD and Rs are the main controlling factors for Ec changes, their effects diminish during the drought period, which aligns with the findings of this study. Chen et al. (2023) and Song et al. (2022) also reported such results. The Ec of these provenances was affected by both the energy and water conditions throughout the study period. In the present study, we observed a correlation between Ec and Ta under drought conditions, which is consistent with the findings of Ouyang et al. (2022) and Tie et al. (2017). Previous studies have also demonstrated that incorporating Ta, Rs, and VPD into transpiration prediction models can improve their accuracy of model predictions (Navarro et al., 2020; Wang et al., 2020). Wang et al. (2022) analyzed the sensitivity of different provenances to climate using tree ring analysis methods, demonstrated that there is the greatest differentiation among Chinese fir provenances occurs in the strength of the correlations between climate variables (temperature and precipitation) and tree ring indices. Furthermore, tree ring width and iWUE are primarily sensitive to temperature (Wang et al., 2024), further confirming the impact of temperature on forest growth and water consumption.

### 4.4 Implications and limitations

Plantations consume large volumes of water to maintain physiological activities (Marchionni et al., 2019). Particularly, during the relatively dry period of June and July 2021, the higher atmospheric evaporation demand promoted the loss of tree water more than in any other month. As the plantation experienced reduced physiological activities from September to March, Ec was maintained at a relatively low level. However, climate change also has a significant effect on water use (Choat et al., 2018). Chinese fir plantations not only ensure timber security but also provides multiple ecosystem services in China’s subtropical region (Zeng et al., 2021), as one of the most effective nature-based solutions (NBS) to mitigate global change. Therefore, it is important to understand the water use and the biophysical governing mechanisms of different provenances to select provenances for afforestation to maximize comprehensive benefits. However, our study focused on the water consumption of provenances from five provinces, which did not provide a complete picture of plant transpiration or the governing mechanisms for all provenances across different climate zones. Provenances collected in a common garden provide an opportunity to comprehensively understand the regulatory mechanisms of tree transpiration and help guide the selection of sustainable forest management objectives for provenances under global climate change.

## 5 Conclusions

This study revealed the difference in canopy transpiration of Chinese fir provenances from five provinces and their unique responses to meteorological factors. The mean daily Ec values of Guangxi, Sichuan, Anhui, Yunan, and Zhejiang provenance were significantly different (p <0.05). Overall, the provenances of Ec were high from June to August. Soil moisture has different effects on the Ec of the provenances. Soil moisture had different effects on the Ec of the provenances. All provenances had higher Ec values when REW <0.4. However, the difference between Guangxi and Yunnan provenances under the two conditions was not significant. When the soil was relatively moist, Ec of the provenances was mainly affected by Rs and VPD. Ta and VPD were the main factors when the soil was relatively dry.

This study highlights the distinct responses of Ec to meteorological conditions among the provenances. The unique response of Ec to varying environments reveals different water use strategies among provenances. Comprehensive insights are needed to understand the complex forest-water relationships and integrated regional forest-water management under the climate change scenario from a water use perspective. The intrinsic physiological mechanisms of plants should also be considered in subsequent research on the factors that affect plant Ec.

## Data Availability

The raw data supporting the conclusions of this article will be made available by the authors, without undue reservation.
